# Identifying Characteristic Frequencies in the Electrochemical Impedance of Ion-Exchange Membrane Systems

**DOI:** 10.3390/membranes12101003

**Published:** 2022-10-16

**Authors:** Antonio Angel Moya

**Affiliations:** Department of Physics, University of Jaén, 23071 Jaén, Spain; aamoya@ujaen.es

**Keywords:** electrochemical impedance, Warburg diffusion impedance, ion-exchange membranes, electric double-layer capacitance, energy storage devices

## Abstract

In this study, the characteristic frequencies of the electrochemical impedance of ion-exchange membrane systems constituted by the membrane and two diffusion boundary layers adjacent to the membrane were investigated. Approximations of the impedance of the Randles equivalent electric circuit in multiple frequency ranges were considered, and the characteristic frequencies of the zeros and poles of orders ½ and 1 were derived. The characteristic geometric frequencies, those associated with the interfacial charge transfer and the diffusive transport processes, as well as those associated with the transitions between processes, were identified by means of analytical expressions.

## 1. Introduction

Studies on electrochemical properties of ion-exchange membrane systems are currently receiving considerable attention due to the potential environmental applications of such systems in various fields of science and technology, such as electrodialysis for brackish water or seawater desalination [1] and renewable energy harvesting, such as pressure-retarded osmosis or reverse electrodialysis [2]. In addition, such membranes are often used as separators in electrochemical energy storage devices, such as rechargeable lithium-ion batteries [3], redox flux batteries [4] and fuel cells [5], or as elements joined to the porous electrodes in systems for capacitive deionization desalination [6] or blue energy production from salinity differences [7].

Electrochemical impedance spectroscopy is a powerful method for characterizing many of the electrical properties of a wide variety of electrochemical systems, including membrane systems [8]. Since the work of Sistat et al. [9], a number of papers dealing with the electrochemical impedance of systems constituted by an ion-exchange membrane and two diffusion boundary layers (DBLs) adjacent to the membrane have appeared in the literature [10,11,12,13,14,15,16,17]. These works include the identification of specific topics in the field of ion exchange, such as diffusion coefficients dependent on ionic concentrations [10], the inhomogeneity of the membrane fixed charge [11], asymmetry of the bathing concentrations [12], geometry of the fluidic channels [13], competitive transport of counter ions [14], water splitting [15], interfacial resistances [16] and fouling [17]. Impedance functions can also be applied in the small-signal dynamic modelling of electrochemical power sources, such as Li-ion batteries and fuel cells, including ion-exchange membranes, which are of particular importance due to the increasing incorporation of such devices into sophisticated electric and electronic systems [18,19]. Multiple studies on the dynamic response of ion-exchange membrane systems have been published in fields related to proton-exchange membrane fuel cells [20,21,22,23].

Impedance experimental data are usually interpreted on the basis of the Randles equivalent electric circuit; the relationship between the circuit elements and the physical and chemical parameters of the system are determined by the Nernst–Planck–Donnan equations under electroneutrality on the basis of the Teorell–Mayer–Sievers model. The differential capacitances of the electric double layers (EDLs) at the membrane–solution interfaces as given by the Gouy–Chapman theory are also included [24]. Although the properties of the Randles circuit model of the electrochemical interface are well-known [25], the characteristic frequencies of the impedance basic function of ion-exchange membrane systems, particularly in the absence of interfacial resistances, have not been investigated in detail.

The main purpose of this paper is to contribute to this area by conducting a novel theoretical study on the identification of the characteristic frequencies of the electrochemical impedance of ion-exchange membrane systems constituted by the membrane and two DBLs adjacent to the membrane on the basis of the zero-pole representation of the approximations of the impedance in multiple frequency ranges. The characteristic frequencies of the zeros and poles of orders ½ and 1 in the impedance of the Randles equivalent electric circuit were derived in this study. The characteristic geometric frequencies, those associated with the interfacial charge transfer and the diffusional transport processes and those associated with the transitions between processes were theoretically identified by means of analytical expressions. The values of these frequencies enabled evaluation of the role played by the capacitances of the EDLs in the impedance model and by the characteristic frequencies within the usual range of experimentation in the characterization of electrical properties of membrane systems.

## 2. Theoretical Basis

**Figure 1:**
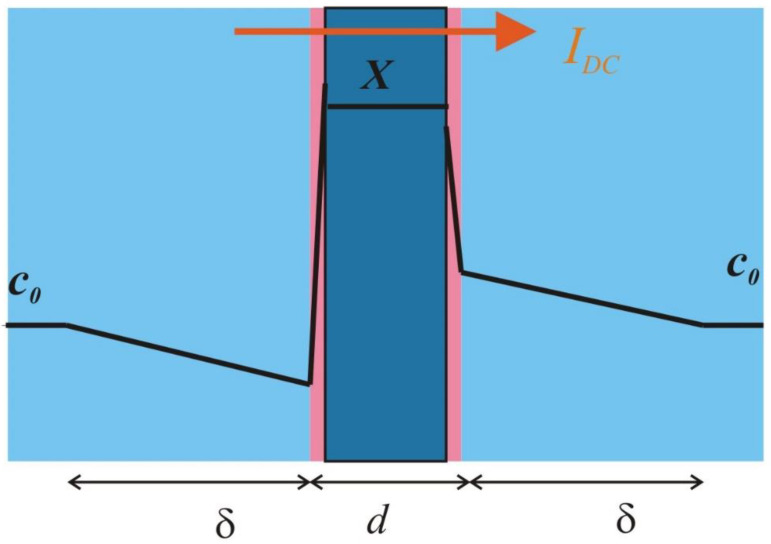
Sketch of the ion-exchange membrane system.

In this study, we considered a membrane with a thickness of *d* and two identical DBLs adjacent to the membrane, each with a thickness of *δ*, as shown in the sketch in Figure 1. The ionic transport is one-dimensional and perpendicular to the membrane–solution interface. The membrane is bathed by two bulk solutions of a 1:1 symmetric binary electrolyte; *c*_0_ is the salt concentration in the solutions, and a cation-exchange membrane with a negative fixed charge of concentration *X* is assumed. The membrane is assumed to be ideal and fully impermeable to co-ions and without interfacial resistance; counter-ion transport is described by means of resistance and geometric capacitance. The diffusion coefficients of the cation and anion in the solution phase are denoted by *D*_1_ and *D*_2_, respectively, and *D_S_* = 2*D*_1_*D*_2_/(*D*_1_ + *D*_2_) is the salt diffusion coefficient. The electric permittivity of the solution phase is denoted by *ε*.

According to the Randles model and previous studies by Nikonenko and Kozmai [24], the impedance of the ion-exchange membrane system, *Z*, can be written as:

(1) $$Z\;=\;Z_{g}\;+\;\frac{1}{s\;C_{DL}\;+\;\frac{1}{Z_{dL}}}\;+\;\frac{1}{s\;C_{DR}\;+\;\frac{1}{Z_{dR}}}$$

where *Z_g_* is the geometric impedance given by:

(2) $$Z_{g}\;=\;\frac{R_{acL}}{1\;+\;s\;R_{acL}\;C_{gL}}\;+\;\frac{R_{M}}{1\;+\;s\;R_{M}\;C_{gM}}\;+\;\frac{R_{acR}}{1\;+\;s\;R_{acR}\;C_{gR}}$$

Here, *s* (*s* = *jω*) is the Laplace variable; *j* is the imaginary unit; and *ω* is the angular frequency, which is 2*π* times the conventional frequency, *f* (*ω* = 2*πf*). *R_M_* and *C_gM_* are the resistance and geometric capacitance of the membrane, respectively. *C_gL_* and *C_gR_* are the geometric capacitances of the DBLs, expressed as:

(3) $$C_{gL}\;=\;C_{gR}\;=\;\frac{\varepsilon }{\delta }$$

The ohmic resistances of the left and right DBLs, respectively, are:

(4) $$R_{acL}\;=\;-\;\frac{R\;T}{F^{2}}\;\frac{\delta }{(D_{1}\;+\;D_{2})\;c_{0}}\;\frac{\ln\;(1\;-\;\beta_{L})}{\beta_{L}}$$

(5) $$R_{acR}\;=\;\frac{R\;T}{F^{2}}\;\frac{\delta }{(D_{1}\;+\;D_{2})\;c_{0}}\;\frac{\ln\;(1\;+\;\beta_{L})}{\beta_{L}}$$

In these expressions, the constants *F*, *R* and *T* represent Faraday constant, ideal gas constant and absolute temperature, respectively. The dimensionless parameter *β_L_* is:

(6) $$\beta_{L}\;=\;\frac{I_{DC}}{I_{L}}$$

i.e., it is the quotient between the dc component of the electric current, *I_DC_*, and the limiting electric current density through the system, *I_L_*, which is obtained when the concentration gradient reaches its maximum value in a DBL or on one of the surfaces of the membrane, expressed as:

(7) $$I_{L}\;=\;\frac{2\;F\;D_{1}\;c_{0}}{\delta }$$

The capacitances of the EDLs at the interfaces are:

(8) $$C_{DL}\;=\;\frac{\varepsilon }{L_{D}}\;\cosh\;\left(\frac{F\;\phi_{DC}^{dL}}{2\;R\;T}\right)$$

(9) $$C_{DR}\;=\;\frac{\varepsilon }{L_{D}}\;\cosh\;\left(\frac{F\;\phi_{DC}^{dR}}{2\;R\;T}\right)$$

where *L_D_* is the Debye length in the solution phases and is expressed as:

(10) $$L_{D}\;=\;\sqrt{\frac{\varepsilon \;R\;T}{2\;F^{2}\;c_{0}}}$$

The Donnan electric potential differences at the solution/membrane interfaces are:

(11) $$\phi_{DC}^{dL}\;=\;\frac{R\;T}{F}\ln\;\left[\frac{X}{c_{0}\;\left(1\;-\;\beta_{L}\right)}\right]\;$$

(12) $$\phi_{DC}^{dR}\;=\;\frac{R\;T}{F}\ln\;\left[\frac{c_{0}\;\left(1\;+\;\beta_{L}\right)}{X}\right]$$

The Warburg diffusion impedances are expressed as:

(13) $$\frac{Z_{dL}}{R_{dL}}\;=\;\frac{Z_{dR}}{R_{dR}}\;=\;\frac{\tanh\;\sqrt{s\;\tau }}{\sqrt{s\;\tau }}$$

where:

(14) $$R_{dL}\;=\;\frac{2\;R\;T}{F\;I_{L}}\;\left(\frac{1}{1\;-\;\beta_{L}}\right)\;-\;R_{acL}$$

(15) $$R_{dR}\;=\;\frac{2\;R\;T}{F\;I_{L}}\;\left(\frac{1}{1\;+\;\beta_{L}}\right)\;-\;R_{acR}$$

and

(16) $$\tau \;=\;\frac{\delta^{2}}{D_{S}}$$

At the highest frequencies, the limit of the impedance function (*Z*) is zero. At intermediate frequencies, the geometric, ohmic or *ac* resistance of the system (*R_ac_*) is the limit of the impedance at the highest frequencies:

(17) $$R_{ac}\;=\;R_{acL}\;+\;R_{M}\;+\;R_{acR}$$

The *DC* resistance of the system (*R_DC_*) is the limit of the impedance at the lowest frequencies:

(18) $$R_{DC}\;=\;R_{M}\;+\;\frac{2\;R\;T}{F\;I_{L}}\;\left(\frac{1}{1\;+\;\beta_{L}}\;+\;\frac{1}{1\;-\;\beta_{L}}\right)$$

The total diffusion resistance is:

(19) $$R_{d}\;=\;R_{dL}\;+\;R_{dR}\;=\;R_{DC}\;-\;R_{ac}$$

**Figure 2:**
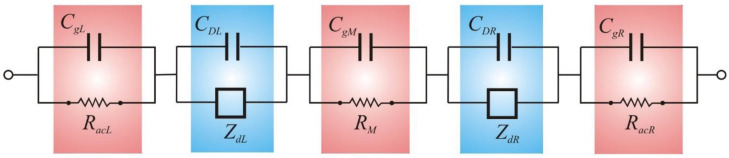
Equivalent electric circuit model of the impedance of an ion-exchange membrane system.

The electrochemical impedance of an ion-exchange membrane system is represented by the electric circuit shown in Figure 2. In the high-frequency range, the impedance is governed by the geometric terms. In the low-frequency range, the Randles circuit at each interface is constituted by the parallel combination of the EDL capacitance and the finite-length Warburg diffusion impedance. The conductive term of the impedance for the non-zero dc component of the electric current, which was originally described by Sistat et al. [9] and included in the model by Nikonenko and Kozmai [24], is ignored. This term appears for dc current values close to the limiting current and does not admit analytical expression, so it is not used to derive the characteristic frequencies. Also, unlike this model [24], the capacitances of the EDLs include the dependence with the Donnan electric potentials.

## 3. Results and Discussion

### 3.1. Impedance Plots

In this study, the electrochemical impedance of an ion-exchange membrane system was numerically obtained from the electric circuit shown in Figure 2 using the electric circuit simulation program PSpice 9.1 (Cadence Design Systems, San Jose, CA, USA) [26]. Simulations were performed for systems with *ε* = 708 pF/m, *δ* = 250 μm, *c*_0_ = 50 mM, *D*_1_ = 1.32·10^−9^ m^2^/s and *D*_2_ = 1.96·10^−9^ m^2^/s. Then, the salt diffusion coefficient is *D_S_* = 1.578·10^−9^ m^2^/s, the Debye length in the solution is *L_D_* = 1.373 nm at *T* = 298 K and the limiting current density is 50.88 A/m^2^. For the membrane, the fixed-charge concentration is *X* = 0.5 M = 100*c*_0_, the resistance is *R_M_* = 0.2 mΩ·m^2^ and the geometric capacitance is *C_gM_* = 2.5 μF/m^2^. The chosen values for the parameters could correspond to typical membranes used in electrodialysis when they are immersed in NaCl-diluted solutions.

**Figure 3:**
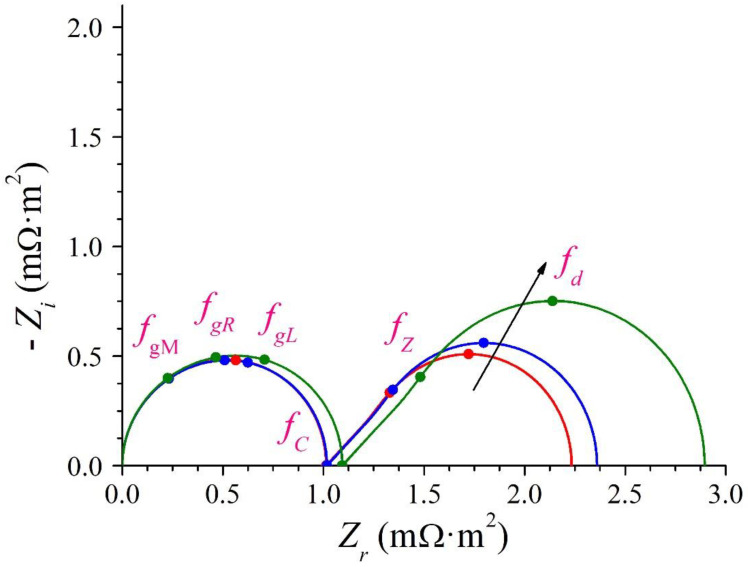
Nyquist plot of the impedance of ion-exchange membrane systems for *I_DC_*/*I_L_* = 0, 0.25 and 0.5. The arrow indicates increasing values of *I_DC_*.

Figure 3 shows the complex-plane impedance plots in a system for *I_DC_* = 0, 0.25*I_L_* and 0.5*I_L_*. The values of the electric parameters corresponding to the circuit shown in Figure 2 are shown in Table 1. In Figure 3, −*Z_i_*(*ω*) is plotted against *Z_r_*(*ω*) with the angular frequency (*ω*) as a parameter increasing from the right to the left of the plot (Nyquist-type plot). This figure shows two regions: a geometric arc at high frequencies in the left of the plot and a diffusion arc at low frequencies in the right of the plot, which is a Warburg-type impedance, as it presents a −45° slope straight line at high frequencies and a semicircle at low frequencies. The geometric arc resembles a distorted semicircle and arises from the superposition of the three semicircles corresponding to the geometric impedances of the membrane and of the two DBLs. The usual experimental Nyquist plots of the impedance of ion-exchange membranes show one arc, in addition to the uncompensated resistance of the system [27], because the geometric arc is only experimentally observed when the measurement instruments operate in the range of very high frequency with values outside the usual range of experimentation from 1 mHz to 1 MHz. Then, the electrical conductivity of ion-exchange membranes system is experimentally determined according to the real part of the impedance at the highest frequencies [28,29].

**Figure 4:**
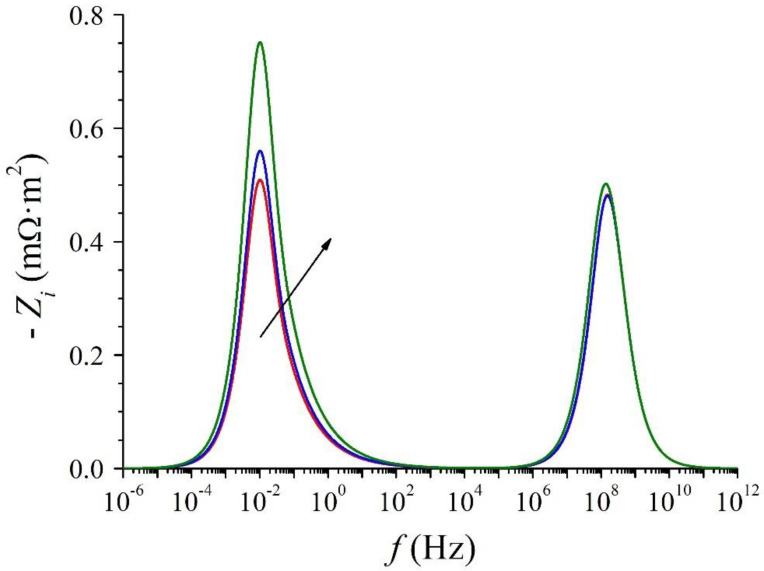
Bode-type plot of the imaginary part of the impedance of ion-exchange membrane systems for *I_DC_*/*I_L_* = 0, 0.25 and 0.5. The arrow indicates increasing values of *I_DC_*.

Figure 4 shows the imaginary part with a minus sign of the impedance (−*Z_i_*) as a function of frequency (*f*) in logarithm scale (Bode-type plot). Two peaks are clearly observed in this figure: a geometric peak at high frequencies and a diffusional peak at low frequencies. Furthermore, the frequency at which the diffusional peak is reached is not a function of the dc component of the electric current as expected [30].

### 3.2. High-Frequency Geometric Impedance

At the highest frequencies, the Warburg diffusion impedance can be approximated by that in semi-infinite space:

(20) $$\frac{Z_{dL}}{R_{dL}}\;=\;\frac{Z_{dR}}{R_{dR}}\;=\;\frac{\tanh\;\sqrt{s\;\tau }}{\sqrt{s\;\tau }}\;\approx \;\frac{1}{\sqrt{s\;\tau }}$$

Then, the impedance of the ion-exchange membrane system at the highest frequencies is:

(21) $$Z_{H}\;=\;Z_{g}\;+\;\frac{R_{dL}}{s\;R_{dL}\;C_{DL}\;+\;\sqrt{s\;\tau }}\;\;+\;\frac{R_{dR}}{s\;R_{dR}\;C_{DR}\;+\;\sqrt{s\;\tau }}$$

The geometric impedance function (*Z_g_*) is a distribution function with three relaxation times. *Z_H_* presents three poles of order 1 at the characteristic frequencies:

(22) $$\omega_{gL}\;=\;2\;\pi \;f_{gL}\;=\;\frac{1}{R_{acL}\;C_{gL}}$$

(23) $$\omega_{gR}\;=\;2\;\pi \;f_{gR}\;=\;\frac{1}{R_{acR}\;C_{gR}}$$

(24) $$\omega_{gM}\;=\;2\;\pi \;f_{gM}\;=\;\frac{1}{R_{M}\;C_{gM}}$$

Moreover, *Z_H_* presents a pole of order ½ at *s* = 0 and two other poles of order ½ with the characteristic frequencies:

(25) $$\omega_{H1}\;=\;2\;\pi \;f_{H1}\;=\;\frac{1}{\tau_{H1}}\;=\;\frac{\tau }{R_{dL}^{2}\;C_{DL}^{2}}$$

(26) $$\omega_{HR}\;=\;2\;\pi \;f_{HR}\;=\;\frac{1}{\tau_{HR}}\;=\;\frac{\tau }{R_{dR}^{2}\;C_{DR}^{2}}$$

Therefore, the impedance at the highest frequencies present five characteristic frequencies corresponding to the membrane, the left and right solutions and the left and right interfaces according to the Teorell–Meyer–Sievers model [31]. Equations (25) and (26) could be considered as the interfacial characteristic frequencies in the absence of interfacial charge transfer resistances [32]. However, the usual values for the EDL capacitances are very small, and the two interfacial frequencies take an extremely high value (of the order of 10^18^ Hz), and they cannot be identified in the impedance plots. By neglecting the EDL capacitances, the impedance of an ion-exchange membrane system at high frequencies is more appropriately expressed as:

(27) $$Z_{H}\;=\;Z_{g}\;+\;\frac{R_{d}}{\sqrt{s\;\tau }}$$

The three geometric frequencies cannot be individually identified. The frequency at which the imaginary part with minus sign of the impedance reaches a maximum in the high-frequency region of the Nyquist (Figure 3) and Bode-type (Figure 4) plots must be numerically obtained based on the expression of *Z_g_*.

On the other hand, the characteristic frequency (*f_C_*) at the intersection point between the geometric and the diffusion arcs in the Nyquist plot or the point at which the imaginary part of the impedance with minus sign reaches a minimum in the overlapped regions of the Nyquist and Bode-type plots can be approximately obtained as follows [33]. Based on a Taylor series at the lowest frequencies for the geometric term and the highest frequencies for the diffusional term, the imaginary part of *Z_H_* in Equation (27) can be expressed as:

(28) $$-\;Z_{Hi}\;=\;\frac{R_{d}}{\sqrt{2\;\omega \;\tau }}\;+\;\left(R_{acL}^{2}\;C_{gL}\;+\;R_{M}^{2}\;C_{gM}\;+\;R_{acR}^{2}\;C_{gR}\right)\;\omega$$

Deriving this function with respect to *ω* and equaling zero, this function reaches a minimum at the frequency (*f_C_*) expressed by the following relation:

(29) $$\omega_{C}\;=\;2\;\pi \;f_{C}\;=\;{\left(\frac{R_{d}}{2\;\left(R_{acL}^{2}\;C_{gL}\;+\;R_{M}^{2}\;C_{gM}\;+\;R_{acR}^{2}\;C_{gR}\right)\;\sqrt{2\;\tau }}\right)}^{\;2/3}$$

### 3.3. Low Frequency Diffusion Impedance

For intermediate frequencies, the impedance corresponding to the Cottrell behavior, which is that obeying a diffusion transport in semi-infinite space, is expressed as:

(30) $$Z_{I}\;=\;R_{ac}\;+\;\frac{R_{d}}{\sqrt{s\;\tau }}$$

This impedance function presents a pole of order ½ at zero frequency. It also presents a zero of order ½ with the following characteristic frequency:

(31) $$\omega_{Z}\;=\;2\;\pi \;f_{Z}\;=\;\frac{R_{d}}{R_{ac}\;\sqrt{\tau }}$$

Table 2 shows the numerical values of the main identified characteristic frequencies. The characteristic frequency above zero is significant in plots related to admittance, i.e., the inverse function of the impedance, but it can be considered the characteristic frequency of the Cottrell behavior in the impedance.

For low frequencies, by ignoring the capacitances of the EDLs, the impedance can be written as:

(32) $$Z_{L}\;=\;R_{ac}\;+\;R_{d}\;\frac{\tanh\;\sqrt{s\;\tau }}{\sqrt{s\;\tau }}$$

According to numerical calculation, the imaginary part with minus sign of *Z_L_* reaches a maximum of value 0.417227*R_d_* at the following frequency:

(33) $$\omega_{d}\;=\;2\;\pi \;f_{d}\;=\;2.54065\;\frac{1}{\tau },$$

which implies the real part, i.e., 0.581634*R_d_*. This frequency is that at which the imaginary part with minus sign of the impedance reaches a maximum in the low-frequency region of the Nyquist (Figure 3) and Bode-type (Figure 4) plots, and it is considered the characteristic frequency of the diffusive transport process in the DBLs. It is also the frequency used to characterize, from an experimental viewpoint, the diffusional behavior in different ion-exchange membrane systems [34].

On the other hand, the frequencies corresponding to the impedance values where the real part, by subtracting *R_ac_*, is equal to minus the imaginary part, i.e., values of the Warburg impedance with a phase of −45°, are expressed as:

(34) $$\;\omega_{dn}\;=\;2\pi \;f_{dn}\;=\;\frac{n^{2}\;\pi^{2}}{2\;\tau }\;,\;\;\;n\;=\;1,\;2,\;3,\;..$$

The transmissive Warburg impedance is constituted by an infinite set of poles corresponding to the Laplace variable values zeroing the denominator. The characteristic frequencies of these poles are expressed as [35]:

(35) $$\omega_{Pn}\;=\;2\;\pi \;f_{Pn}\;=\;{\left[\frac{\pi \;(2\;n\;-\;1)}{2}\right]}^{2}\frac{1}{\tau }\;,\;\;\;n\;=\;1,\;2,\;3,\;..$$

Then, *Z_L_* can be developed into partial fractions by means of Foster series as [35]:

(36) $$Z_{L}\;=\;R_{ac}\;+\;\frac{2\;R_{d}}{\tau }\;\sum_{n\;=\;1}^{\infty }\;\frac{\tau_{Pn}}{1\;+\;s\;\tau_{Pn}}$$

The Taylor series around *s* = 0 of the transmissive Warburg impedance is:

(37) $$Z_{L}\;=\;R_{DC}\;-\;\frac{R_{d}}{3}\;s\;\tau \;+\;\frac{2\;R_{d}}{15}\;{\left(s\;\tau \right)}^{2}\;-\;\frac{17\;R_{d}}{315}\;{\left(s\;\tau \right)}^{3}\;+\;O\;(s^{4})$$

Operating with the inverse function of the admittance with successive continued fractions yields [35]:

(38) $$Z_{L}\;=\;R_{ac}\;+\;\frac{1}{\frac{1}{R_{d}}\;+\;\frac{1}{\frac{3\;R_{d}}{s\;\tau }\;+\;\frac{1}{\frac{5}{R_{d}}\;+\;\ldots }}}$$

At very low frequencies, by considering only the first pole of the transmissive Warburg impedance, the impedance can be expressed as:

(39) $$Z_{F1}\;=\;R_{ac}\;+\;\frac{R_{d}}{1\;+\;s\;\frac{4\;\tau }{\pi^{2}}},$$

which presents the characteristic frequency corresponding to the first pole of the Warburg impedance:

(40) $$\omega_{P1}\;=\;2\;\pi \;f_{P1}\;=\;\frac{\pi^{2}}{4\;\tau }$$

Moreover, at the lowest frequencies, the Taylor series with two terms yields:

(41) $$Z_{T1}\;=\;R_{ac}\;+\;\frac{R_{d}}{1\;+\;s\;\frac{\tau }{3}},$$

which yields the characteristic frequency:

(42) $$\omega_{T1}\;=\;2\;\pi \;f_{T1}\;=\;3\;\frac{1}{\tau }$$

At very low frequencies, the Foster series of the impedance can be expressed as:

(43) $$Z_{F}\;=\;R_{ac}\;+\;\left(1\;-\;\frac{8}{\pi^{2}}\right)\;R_{d}\;+\;\frac{\frac{8}{\pi^{2}}\;R_{d}}{1\;+\;s\;\frac{4\;\tau }{\pi^{2}}},$$

which presents the frequency of the first pole of the Warburg impedance given by Equation (40) as the characteristic frequency. Finally, at the lowest frequencies, the Taylor series with three elements [36] is expressed as:

(44) $$Z_{T}\;=\;R_{ac}\;+\;\frac{R_{d}}{6}\;+\;\frac{5}{6}\;\frac{R_{d}}{1\;+\;s\;\frac{2\;\tau }{5}}$$

which yields the characteristic frequency:

(45) $$\omega_{T}\;=\;2\;\pi \;f_{T}\;=\;2.5\;\frac{1}{\tau }$$

**Figure 5:**
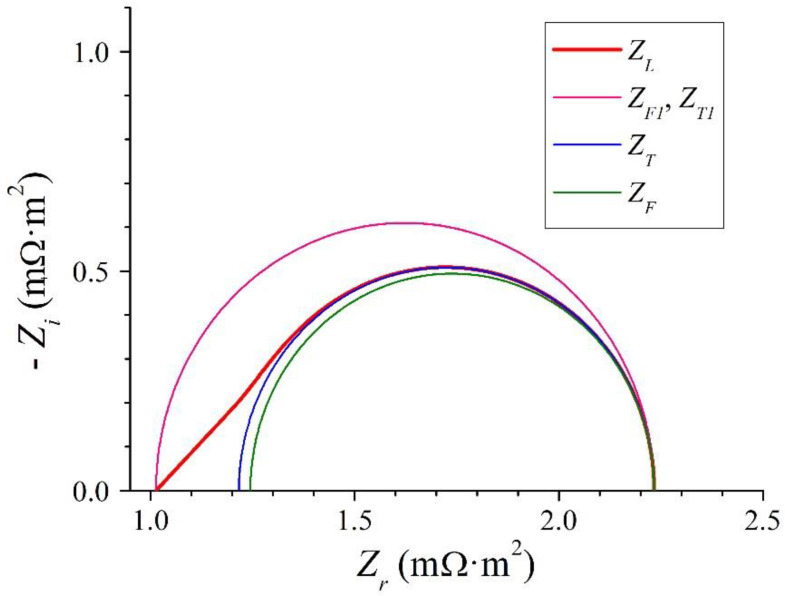
Nyquist plots of the impedance of ion-exchange membrane systems at low frequencies for *I_DC_* = 0 and the approximated impedances obtained according to developments of the Warburg impedance.

The Nyquist plot of the low-frequency impedance of ion-exchange membrane systems (*Z_L_*) and those of the different approximations corresponding to the lowest frequencies (*Z_F_*_1_, *Z_T_*_1_, *Z_F_* and *Z_T_*) are shown in Figure 5 for *I_DC_* = 0. The approximation obtained using continued fractions with three terms from the Taylor series around zero frequency of the Warburg impedance appropriately models the impedance of ion-exchange membrane systems at low frequencies [36]. The corresponding characteristic frequency expressed by Equation (45) is very close to the characteristic frequency of the diffusive transport process expressed by Equation (33).

## 4. Conclusions

A set of characteristic frequencies of the electrochemical impedance of ion-exchange membrane systems were identified from the zero-pole representation of the approximations of high and low frequencies. This approach allows us to use the values of these frequencies within the range of experimentation for the characterization of electrical properties of ion-exchange membrane systems. In addition to the characteristic geometric frequencies in the membrane and the two DBLs adjacent to the membrane, we identified two new characteristic interfacial frequencies. These frequencies correspond to poles of order ½ of the impedance associated with the contributions of the EDLs, and their numerical values allow us to evaluate the role played by the capacitances of the EDLs in the impedance of ion-exchange membrane systems without interfacial resistances. In the intermediate frequency range, a new characteristic frequency associated with the Cottrell behavior of the system obeying a diffusion transport in semi-infinite space was identified, which would be relevant in studies associated to admittance. In the low frequency range, multiple frequencies were identified, in addition to the characteristic diffusional frequency, i.e., that corresponding to the maximum of the imaginary part with minus sign of the impedance. These are the characteristic frequencies of the infinite poles of the hyperbolic tangent function and those at which the phase of this function is −45°. At very low frequencies, the impedance can be modelled by a function with a single frequency. The expansion into partial fractions of the Warburg impedance exhibits the frequency of the first pole as the characteristic frequency, whereas two additional expressions for the characteristic frequency associated with the expansions in Taylor series can also be identified.

## Figures and Tables

**Table 1:** Parameters of the electric circuit shown in Figure 2.

	*I_DC_* = 0	*I_DC_* = 0.25 *I_L_*	*I_DC_* = 0.5 *I_L_*
*R_acL_* (mΩ·m^2^)	0.4067	0.4603	0.5638
*R_acR_* (mΩ·m^2^)	0.4067	0.3570	0.3298
*R_M_* (mΩ·m^2^)	0.2		
*C_gL_* (μF/m^2^)	2.832		
*C_gR_* (μF/m^2^)	2.832		
*C_gM_* (μF/m^2^)	2.5		
*R_dL_* (mΩ·m^2^)	0.61	0.9407	1.4573
*R_dR_* (mΩ·m^2^)	0.61	0.4017	0.3439
*C_DL_* (μF/m^2^)	2.602	2.997	3.6618
*C_DR_* (μF/m^2^)	2.602	2.333	2.1352
*τ* (s)	39.617		

**Table 2:** Numerical values of the main characteristic frequencies in an ion-exchange membrane system.

	*I_DC_* = 0	*I_DC_* = 0.25 *I_L_*	*I_DC_* = 0.5 *I_L_*
*f_gL_* (Hz)	140.5 × 10^6^	122.1 × 10^6^	99.68 × 10^6^
*f_gR_* (Hz)	140.5 × 10^6^	157.4 × 10^6^	170.4 × 10^6^
*f_gM_* (Hz)	318.3 × 10^6^		
*f_HL_* (Hz)	2.51 × 10^18^	0.793 × 10^18^	0.221 × 10^18^
*f_HR_* (Hz)	2.51 × 10^18^	7.18 × 10^18^	11.69 × 10^18^
*f_C_* (Hz)	25.9 × 10^3^	27.3 × 10^3^	28.9 × 10^3^
*f_Z_* (Hz)	0.03044	0.03337	0.04165
*f_d_* (Hz)	0.01021		

## Data Availability

Not applicable.
